# (*E*,*E*)-2,2′-[1,1′-(Cyclohexane-1,2-diyl­dinitrilo)diethylidyne]diphenol

**DOI:** 10.1107/S160053680802552X

**Published:** 2008-08-16

**Authors:** Fang Chen, Heng-Yun Ye

**Affiliations:** a Ordered Matter Science Research Center, College of Chemistry and Chemical Engineering, Southeast University, Nanjing 210096, People’s Republic of China

## Abstract

The title compound, C_22_H_26_N_2_O_2_, is chiral; the absolute configuration follows from the known chirality of the input reagents. The asymmetric unit contains two crystallographically independent mol­ecules in different orientations. The two mol­ecules are related to each other by a non-crystallographic twofold rotation axis, while each mol­ecule exhibits a further pseudo-twofold axis. Bond distances and angles are similar in the two mol­ecules. Inter­molecular C—H⋯π(ring) inter­actions and intra­molecular O—H⋯N hydrogen bonds are observed in the crystal structure.

## Related literature

For examples of syntheses of non-centrosymmetric solid materials by reaction of chiral organic ligands and inorganic salts, see: Qu *et al.* (2004 ▶). For related structures, see: Figuet *et al.* (2001 ▶); Kennedy & Reglinski (2001 ▶); Thamotharan *et al.* (2003 ▶).
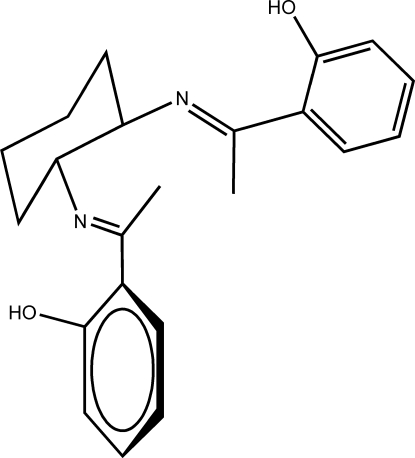


## Experimental

### 

#### Crystal data

C_22_H_26_N_2_O_2_
*M* *_r_* = 350.45
Monoclinic, 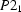

*a* = 12.608 (3) Å
*b* = 11.185 (2) Å
*c* = 14.438 (3) Å
β = 106.14 (3)°
*V* = 1955.8 (8) Å^3^
*Z* = 4
Mo *K*α radiation
μ = 0.08 mm^−1^
*T* = 293 (2) K
0.25 × 0.15 × 0.15 mm

#### Data collection

Rigaku SCXmini diffractometer
Absorption correction: multi-scan (*CrystalClear*; Rigaku, 2005 ▶) *T*_min_ = 0.839, *T*_max_ = 1.000 (expected range = 0.829–0.989)
20307 measured reflections
4712 independent reflections
2978 reflections with *I* > 2σ(*I*)
*R*_int_ = 0.079

#### Refinement

*R*[*F* ^2^ > 2σ(*F* ^2^)] = 0.057
*wR*(*F* ^2^) = 0.134
*S* = 1.04
4712 reflections
473 parameters
1 restraint
H-atom parameters constrained
Δρ_max_ = 0.15 e Å^−3^
Δρ_min_ = −0.21 e Å^−3^

### 

Data collection: *CrystalClear* (Rigaku, 2005 ▶); cell refinement: *CrystalClear*; data reduction: *CrystalClear*; program(s) used to solve structure: *SHELXS97* (Sheldrick, 2008 ▶); program(s) used to refine structure: *SHELXL97* (Sheldrick, 2008 ▶); molecular graphics: *SHELXTL* (Sheldrick, 2008 ▶); software used to prepare material for publication: *SHELXTL*.

## Supplementary Material

Crystal structure: contains datablocks I, global. DOI: 10.1107/S160053680802552X/ez2132sup1.cif

Structure factors: contains datablocks I. DOI: 10.1107/S160053680802552X/ez2132Isup2.hkl

Additional supplementary materials:  crystallographic information; 3D view; checkCIF report

## Figures and Tables

**Table 1:** Selected torsion angles (°)

N1—C1—C6—N2	−69.1 (3)
N3—C28—C23—N4	−65.9 (3)

**Table 2:** Hydrogen-bond geometry (Å, °)

*D*—H⋯*A*	*D*—H	H⋯*A*	*D*⋯*A*	*D*—H⋯*A*
O1—H1⋯N1	0.82	1.77	2.496 (4)	147
O2—H2⋯N2	0.82	1.81	2.531 (4)	147
O3—H3⋯N3	0.82	1.82	2.507 (4)	140
O4—H4⋯N4	0.82	1.78	2.507 (4)	147
C26—H26*A*⋯*Cg*3^i^	0.97	2.96	3.790 (5)	144
C29—H29*C*⋯*Cg*3^ii^	0.96	2.96	3.721 (5)	137
C37—H37*C*⋯*Cg*3^iii^	0.96	3.00	3.714 (4)	133

Symmetry codes: (i) 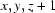; (ii) 
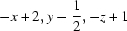
; (iii) 
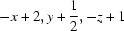
. *Cg*3 is the centroid of the C17–C22 ring and *Cg*2 is the centroid of the C9–C14 ring.

**Fig. 1.**
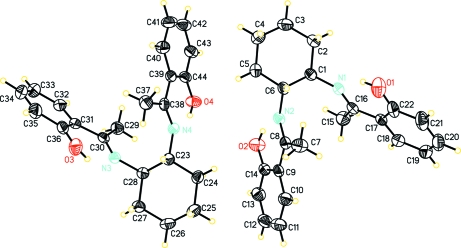
A view of the title compound with the atomic numbering scheme. Displacement ellipsoids are drawn at the 30% probability level.

## supplementary crystallographic information

### Comment

The existence of a chiral centre in an organic ligand is very important for the construction of noncentrosymmetric or chiral coordination polymers that exhibit desirable physical properties such as ferroelectric behavior (Qu *et al.*, 2004). As a part of our ongoing investigations in this field we have determined the crystal structure of the title compound, (I).

Fig. 1 shows the asymmetric unit consisting of two molecules of (I). The two crystallographically independent molecules have the same geometrical parameters within the precision of the experiments. The bond lengths and angles in (I) are comparable to the corresponding values in the related structures, tris[(5-bromosalicylidene)aminoethyl]amine (Figuet *et al.*, 2001), *N*,*N*'-bis(salicylidene)-1,4-butanediamine (Kennedy & Reglinski, 2001) and *N*-(4-Butylphenyl)salicylaldimine (Thamotharan *et al.*, 2003). The average for the N1—C1—C6—N2 and N3—C28—C23—N4 torsion angles is 67.5 (3)°, the average dihedral angle between two benzene rings within one molecule is 48.0 (1)°, and the average distance between the centers of the two benzene rings is 6.53 Å. Like other Schiff base compounds containing salicylidene (Figuet *et al.*, 2001; Kennedy & Reglinski, 2001; Thamotharan *et al.*, 2003) the hydroxyl groups form intramolecular hydrogen bonds with the N atoms (Table 2), thereby completing six-membered rings.

### Experimental

*o*-Hydroxyacetophenone (0.68 g, 5.0 mmol) and (1*R*,2*R*)-(-)-diaminocyclohexane (0.30 g, 2.6 mmol) were dissolved in ethanol (30 mL), and heated to reflux for 8 h until the raw material disappeared according to TLC detection. The solution was cooled to room temperature, then solvent was removed under reduced pressure. The residue was recrystallized with iso-propanol to afford yellow crystals, some of which were suitable for X-ray analysis.

### Refinement

Positional parameters of the H atoms bonded to C atoms were calculated geometrically and were allowed to ride on the C atoms with C_methine_—H_methine _= 0.97; C_methylene_—H_methylene _= 0.96; C_aryl_—H_aryl_=0.93 Å; *U*_iso_H = 1.2 *U*_eq_C. Positional parameters of the H atoms bonded to O atoms were calculated geometrically with the C—O—H angle tetrahedtral and refined in a rotating mode with O—H = 0.82 Å and *U*_iso_H = 1.5 *U*_eq_O. In the absence of significant anomalous scattering effects, 4211 Friedel pairs were merged.

### Crystal data

**Table:**

C_22_H_26_N_2_O_2_	*F*_000_ = 752
*M**_r_* = 350.45	*D*_x_ = 1.190 Mg m^−^^3^
Monoclinic, *P*2_1_	Mo *K*α radiation λ = 0.71073 Å
Hall symbol: P 2yb	Cell parameters from 15855 reflections
*a* = 12.608 (3) Å	θ = 3.1–27.5º
*b* = 11.185 (2) Å	µ = 0.08 mm^−^^1^
*c* = 14.438 (3) Å	*T* = 293 (2) K
β = 106.14 (3)º	Block, yellow
*V* = 1955.8 (8) Å^3^	0.25 × 0.15 × 0.15 mm
*Z* = 4	

### Data collection

**Table:**

Rigaku SCXmini diffractometer	4712 independent reflections
Radiation source: fine-focus sealed tube	2978 reflections with *I* > 2σ(*I*)
Monochromator: graphite	*R*_int_ = 0.079
Detector resolution: 13.6612 pixels mm^-1^	θ_max_ = 27.5º
*T* = 293(2) K	θ_min_ = 3.1º
ω scans	*h* = −16→16
Absorption correction: multi-scan(CrystalClear; Rigaku, 2005)	*k* = −14→14
*T*_min_ = 0.839, *T*_max_ = 1.000	*l* = −18→18
20307 measured reflections	

### Refinement

**Table:**

Refinement on *F*^2^	Secondary atom site location: difference Fourier map
Least-squares matrix: full	Hydrogen site location: inferred from neighbouring sites
*R*[*F*^2^ > 2σ(*F*^2^)] = 0.057	H-atom parameters constrained
*wR*(*F*^2^) = 0.134	*w* = 1/[σ^2^(*F*_o_^2^) + (0.0522*P*)^2^ + 0.0566*P*] where *P* = (*F*_o_^2^ + 2*F*_c_^2^)/3
*S* = 1.04	(Δ/σ)_max_ < 0.001
4712 reflections	Δρ_max_ = 0.15 e Å^−^^3^
473 parameters	Δρ_min_ = −0.21 e Å^−^^3^
1 restraint	Extinction correction: none
Primary atom site location: structure-invariant direct methods	

### Special details

**Table:**

Geometry. All e.s.d.'s (except the e.s.d. in the dihedral angle between two l.s. planes) are estimated using the full covariance matrix. The cell e.s.d.'s are taken into account individually in the estimation of e.s.d.'s in distances, angles and torsion angles; correlations between e.s.d.'s in cell parameters are only used when they are defined by crystal symmetry. An approximate (isotropic) treatment of cell e.s.d.'s is used for estimating e.s.d.'s involving l.s. planes.
Refinement. Refinement of *F*^2^ against ALL reflections. The weighted *R*-factor *wR* and goodness of fit *S* are based on *F*^2^, conventional *R*-factors *R* are based on *F*, with *F* set to zero for negative *F*^2^. The threshold expression of *F*^2^ > σ(*F*^2^) is used only for calculating *R*-factors(gt) *etc*. and is not relevant to the choice of reflections for refinement. *R*-factors based on *F*^2^ are statistically about twice as large as those based on *F*, and *R*- factors based on ALL data will be even larger.

### Fractional atomic coordinates and isotropic or equivalent isotropic displacement parameters (Å^2^)

**Table:**

	*x*	*y*	*z*	*U*_iso_*/*U*_eq_	
C1	0.8617 (3)	0.4943 (3)	0.1556 (2)	0.0489 (8)	
H1A	0.8633	0.4067	0.1563	0.059*	
C2	0.7600 (3)	0.5372 (4)	0.0777 (2)	0.0653 (10)	
H2A	0.7632	0.6234	0.0719	0.078*	
H2B	0.7606	0.5025	0.0163	0.078*	
C3	0.6538 (3)	0.5033 (5)	0.1001 (3)	0.0859 (14)	
H3A	0.6471	0.4169	0.1004	0.103*	
H3B	0.5916	0.5347	0.0505	0.103*	
C4	0.6516 (3)	0.5525 (5)	0.1977 (3)	0.0810 (13)	
H4A	0.6516	0.6392	0.1958	0.097*	
H4B	0.5847	0.5266	0.2124	0.097*	
C5	0.7509 (3)	0.5092 (4)	0.2750 (2)	0.0621 (10)	
H5A	0.7462	0.4230	0.2806	0.075*	
H5B	0.7496	0.5440	0.3362	0.075*	
C6	0.8595 (3)	0.5404 (3)	0.2551 (2)	0.0481 (8)	
H6A	0.8692	0.6274	0.2576	0.058*	
C7	1.0699 (3)	0.6613 (3)	0.3580 (3)	0.0662 (11)	
H7A	1.0117	0.6972	0.3082	0.099*	
H7B	1.1367	0.6607	0.3385	0.099*	
H7C	1.0812	0.7066	0.4164	0.099*	
C8	1.0387 (3)	0.5365 (3)	0.3748 (2)	0.0468 (8)	
C9	1.1180 (3)	0.4648 (3)	0.4499 (2)	0.0469 (8)	
C10	1.2200 (3)	0.5109 (4)	0.5008 (3)	0.0676 (11)	
H10A	1.2390	0.5874	0.4858	0.081*	
C11	1.2940 (4)	0.4480 (5)	0.5724 (3)	0.0806 (13)	
H11A	1.3615	0.4814	0.6054	0.097*	
C12	1.2654 (4)	0.3336 (5)	0.5943 (3)	0.0785 (13)	
H12A	1.3142	0.2894	0.6421	0.094*	
C13	1.1657 (3)	0.2861 (4)	0.5457 (3)	0.0676 (11)	
H13A	1.1476	0.2094	0.5610	0.081*	
C14	1.0911 (3)	0.3494 (3)	0.4743 (2)	0.0517 (9)	
C15	1.0654 (3)	0.3516 (3)	0.1656 (3)	0.0702 (11)	
H15A	1.0023	0.3225	0.1835	0.105*	
H15B	1.1298	0.3456	0.2197	0.105*	
H15C	1.0759	0.3046	0.1132	0.105*	
C16	1.0473 (3)	0.4797 (3)	0.1349 (2)	0.0478 (8)	
C17	1.1339 (3)	0.5435 (4)	0.1040 (2)	0.0512 (9)	
C18	1.2338 (3)	0.4887 (5)	0.1053 (3)	0.0716 (11)	
H18A	1.2457	0.4098	0.1260	0.086*	
C19	1.3151 (4)	0.5479 (7)	0.0767 (3)	0.0967 (18)	
H19A	1.3808	0.5093	0.0779	0.116*	
C20	1.2980 (5)	0.6670 (7)	0.0457 (3)	0.0980 (19)	
H20A	1.3528	0.7088	0.0277	0.118*	
C21	1.2004 (5)	0.7204 (5)	0.0424 (3)	0.0856 (14)	
H21A	1.1886	0.7985	0.0197	0.103*	
C22	1.1184 (4)	0.6632 (4)	0.0713 (3)	0.0643 (11)	
C23	0.8857 (2)	0.5409 (3)	0.6760 (2)	0.0444 (7)	
H23A	0.8790	0.6268	0.6868	0.053*	
C24	0.9936 (3)	0.5162 (4)	0.6505 (2)	0.0539 (9)	
H24A	0.9959	0.4328	0.6328	0.065*	
H24B	0.9960	0.5644	0.5953	0.065*	
C25	1.0939 (3)	0.5445 (4)	0.7343 (3)	0.0656 (10)	
H25A	1.1604	0.5230	0.7170	0.079*	
H25B	1.0963	0.6297	0.7471	0.079*	
C26	1.0910 (3)	0.4776 (4)	0.8245 (3)	0.0653 (10)	
H26A	1.1530	0.5025	0.8775	0.078*	
H26B	1.0985	0.3926	0.8145	0.078*	
C27	0.9838 (3)	0.5003 (3)	0.8508 (2)	0.0536 (9)	
H27A	0.9808	0.5836	0.8687	0.064*	
H27B	0.9825	0.4517	0.9061	0.064*	
C28	0.8828 (2)	0.4711 (3)	0.7670 (2)	0.0442 (8)	
H28A	0.8817	0.3852	0.7535	0.053*	
C29	0.6789 (3)	0.3226 (4)	0.7262 (3)	0.0618 (10)	
H29A	0.7456	0.3005	0.7108	0.093*	
H29B	0.6198	0.3290	0.6678	0.093*	
H29C	0.6609	0.2627	0.7670	0.093*	
C30	0.6950 (3)	0.4407 (3)	0.7775 (2)	0.0460 (8)	
C31	0.6028 (3)	0.4925 (4)	0.8083 (2)	0.0537 (9)	
C32	0.5077 (3)	0.4256 (5)	0.8044 (3)	0.0759 (13)	
H32A	0.5043	0.3459	0.7854	0.091*	
C33	0.4196 (4)	0.4758 (7)	0.8280 (3)	0.0983 (18)	
H33A	0.3579	0.4293	0.8263	0.118*	
C34	0.4212 (4)	0.5939 (7)	0.8541 (3)	0.0988 (19)	
H34A	0.3598	0.6283	0.8672	0.119*	
C35	0.5133 (4)	0.6599 (5)	0.8605 (3)	0.0827 (14)	
H35A	0.5146	0.7396	0.8793	0.099*	
C36	0.6064 (3)	0.6113 (4)	0.8396 (2)	0.0608 (10)	
C37	0.6766 (3)	0.6765 (3)	0.6016 (3)	0.0625 (10)	
H37A	0.7382	0.7020	0.6534	0.094*	
H37B	0.6128	0.6671	0.6250	0.094*	
H37C	0.6616	0.7353	0.5512	0.094*	
C38	0.7033 (3)	0.5594 (3)	0.5629 (2)	0.0458 (8)	
C39	0.6193 (3)	0.5043 (3)	0.4824 (2)	0.0466 (8)	
C40	0.5179 (3)	0.5605 (4)	0.4382 (3)	0.0658 (10)	
H40A	0.5032	0.6346	0.4611	0.079*	
C41	0.4406 (3)	0.5103 (5)	0.3628 (3)	0.0759 (13)	
H41A	0.3746	0.5501	0.3352	0.091*	
C42	0.4603 (3)	0.4007 (4)	0.3276 (3)	0.0713 (12)	
H42A	0.4079	0.3665	0.2760	0.086*	
C43	0.5573 (3)	0.3421 (4)	0.3689 (3)	0.0624 (10)	
H43A	0.5701	0.2680	0.3449	0.075*	
C44	0.6369 (3)	0.3913 (3)	0.4457 (2)	0.0508 (9)	
N1	0.9588 (2)	0.5395 (3)	0.13073 (17)	0.0476 (7)	
N2	0.9485 (2)	0.4849 (2)	0.32967 (17)	0.0463 (7)	
N3	0.7838 (2)	0.5031 (2)	0.79504 (17)	0.0459 (6)	
N4	0.7941 (2)	0.5016 (2)	0.59532 (18)	0.0452 (6)	
O1	1.0250 (3)	0.7226 (2)	0.0652 (2)	0.0832 (9)	
H1	0.9804	0.6779	0.0794	0.125*	
O2	0.9943 (2)	0.2974 (2)	0.4311 (2)	0.0733 (8)	
H2	0.9589	0.3405	0.3875	0.110*	
O3	0.6953 (2)	0.6804 (2)	0.8496 (2)	0.0701 (7)	
H3	0.7488	0.6384	0.8503	0.105*	
O4	0.7305 (2)	0.3302 (2)	0.48161 (19)	0.0667 (7)	
H4	0.7726	0.3696	0.5242	0.100*	

### Atomic displacement parameters (Å^2^)

**Table:**

	*U*^11^	*U*^22^	*U*^33^	*U*^12^	*U*^13^	*U*^23^
C1	0.0546 (19)	0.0462 (19)	0.0449 (17)	0.0008 (17)	0.0119 (15)	0.0042 (16)
C2	0.062 (2)	0.083 (3)	0.0469 (19)	0.001 (2)	0.0093 (17)	0.008 (2)
C3	0.057 (2)	0.120 (4)	0.069 (3)	−0.006 (3)	−0.0028 (19)	0.017 (3)
C4	0.055 (2)	0.107 (4)	0.083 (3)	0.006 (3)	0.023 (2)	0.020 (3)
C5	0.061 (2)	0.074 (3)	0.055 (2)	−0.002 (2)	0.0225 (18)	0.003 (2)
C6	0.0548 (19)	0.0464 (19)	0.0446 (18)	−0.0062 (17)	0.0161 (15)	0.0042 (16)
C7	0.068 (2)	0.048 (2)	0.073 (2)	−0.016 (2)	0.004 (2)	0.008 (2)
C8	0.0545 (19)	0.0467 (19)	0.0418 (17)	−0.0036 (17)	0.0178 (15)	−0.0025 (15)
C9	0.056 (2)	0.050 (2)	0.0377 (17)	−0.0027 (17)	0.0176 (15)	−0.0051 (15)
C10	0.066 (2)	0.073 (3)	0.058 (2)	−0.011 (2)	0.0095 (19)	0.003 (2)
C11	0.068 (3)	0.100 (4)	0.062 (3)	−0.007 (3)	−0.003 (2)	0.001 (3)
C12	0.077 (3)	0.096 (4)	0.056 (2)	0.024 (3)	0.008 (2)	0.009 (2)
C13	0.078 (3)	0.060 (2)	0.063 (2)	0.012 (2)	0.016 (2)	0.011 (2)
C14	0.056 (2)	0.054 (2)	0.0447 (19)	0.0023 (18)	0.0143 (16)	0.0006 (17)
C15	0.074 (3)	0.055 (2)	0.082 (3)	0.012 (2)	0.024 (2)	0.013 (2)
C16	0.062 (2)	0.047 (2)	0.0317 (15)	0.0017 (18)	0.0088 (15)	0.0032 (14)
C17	0.053 (2)	0.065 (2)	0.0331 (16)	−0.0066 (19)	0.0089 (15)	−0.0019 (16)
C18	0.058 (2)	0.099 (3)	0.055 (2)	0.001 (2)	0.0117 (18)	0.008 (2)
C19	0.061 (3)	0.171 (6)	0.058 (3)	−0.021 (4)	0.016 (2)	−0.007 (3)
C20	0.093 (4)	0.152 (6)	0.055 (3)	−0.064 (4)	0.032 (3)	−0.020 (3)
C21	0.116 (4)	0.082 (3)	0.069 (3)	−0.041 (3)	0.043 (3)	−0.010 (2)
C22	0.090 (3)	0.060 (2)	0.051 (2)	−0.017 (2)	0.032 (2)	−0.0024 (19)
C23	0.0459 (18)	0.0395 (17)	0.0475 (17)	0.0024 (16)	0.0125 (14)	−0.0082 (15)
C24	0.0498 (19)	0.056 (2)	0.059 (2)	0.0010 (17)	0.0201 (16)	−0.0061 (18)
C25	0.0452 (19)	0.072 (3)	0.079 (3)	−0.003 (2)	0.0167 (19)	−0.011 (2)
C26	0.044 (2)	0.068 (3)	0.073 (3)	0.0002 (19)	−0.0009 (17)	−0.009 (2)
C27	0.0524 (19)	0.054 (2)	0.0497 (19)	0.0043 (18)	0.0061 (15)	−0.0026 (17)
C28	0.0467 (18)	0.0364 (17)	0.0477 (18)	0.0043 (15)	0.0101 (14)	−0.0009 (15)
C29	0.062 (2)	0.065 (2)	0.058 (2)	−0.011 (2)	0.0145 (18)	−0.008 (2)
C30	0.0482 (19)	0.053 (2)	0.0339 (16)	0.0023 (17)	0.0061 (14)	0.0014 (15)
C31	0.0443 (19)	0.078 (3)	0.0359 (16)	0.0059 (19)	0.0062 (14)	0.0046 (18)
C32	0.048 (2)	0.124 (4)	0.054 (2)	−0.007 (2)	0.0105 (18)	−0.009 (2)
C33	0.055 (3)	0.173 (6)	0.071 (3)	−0.008 (3)	0.023 (2)	−0.012 (4)
C34	0.062 (3)	0.175 (6)	0.065 (3)	0.035 (4)	0.026 (2)	0.001 (4)
C35	0.083 (3)	0.107 (4)	0.062 (3)	0.036 (3)	0.026 (2)	0.005 (3)
C36	0.062 (2)	0.083 (3)	0.0400 (19)	0.019 (2)	0.0178 (18)	0.0066 (19)
C37	0.071 (2)	0.059 (2)	0.058 (2)	0.025 (2)	0.0190 (19)	0.0029 (19)
C38	0.0503 (19)	0.049 (2)	0.0440 (17)	0.0090 (17)	0.0227 (15)	0.0068 (16)
C39	0.0457 (18)	0.053 (2)	0.0419 (17)	0.0040 (16)	0.0143 (14)	0.0109 (16)
C40	0.056 (2)	0.076 (3)	0.063 (2)	0.008 (2)	0.0127 (19)	0.011 (2)
C41	0.050 (2)	0.091 (4)	0.076 (3)	0.005 (2)	0.000 (2)	0.026 (3)
C42	0.058 (3)	0.083 (3)	0.063 (3)	−0.019 (2)	0.001 (2)	0.019 (2)
C43	0.063 (2)	0.065 (2)	0.054 (2)	−0.017 (2)	0.0075 (19)	0.0057 (19)
C44	0.051 (2)	0.055 (2)	0.0428 (19)	−0.0020 (18)	0.0078 (16)	0.0073 (17)
N1	0.0564 (16)	0.0468 (16)	0.0398 (14)	−0.0001 (15)	0.0136 (12)	0.0050 (13)
N2	0.0533 (16)	0.0438 (16)	0.0407 (14)	−0.0073 (14)	0.0112 (13)	0.0029 (13)
N3	0.0463 (15)	0.0470 (16)	0.0439 (14)	0.0026 (14)	0.0117 (12)	−0.0042 (13)
N4	0.0436 (15)	0.0464 (16)	0.0454 (15)	0.0068 (14)	0.0121 (12)	−0.0005 (13)
O1	0.115 (3)	0.0492 (17)	0.101 (2)	0.0006 (18)	0.057 (2)	0.0104 (16)
O2	0.0760 (18)	0.0524 (16)	0.0798 (19)	−0.0119 (15)	0.0023 (14)	0.0146 (14)
O3	0.0811 (19)	0.0616 (17)	0.0761 (18)	0.0126 (15)	0.0362 (16)	−0.0051 (15)
O4	0.0641 (16)	0.0548 (15)	0.0686 (17)	0.0047 (14)	−0.0023 (13)	−0.0095 (13)

### Geometric parameters (Å, °)

**Table:**

C1—N1	1.459 (4)	C23—C28	1.538 (4)
C1—C2	1.529 (4)	C23—H23A	0.9800
C1—C6	1.534 (4)	C24—C25	1.521 (5)
C1—H1A	0.9800	C24—H24A	0.9700
C2—C3	1.511 (5)	C24—H24B	0.9700
C2—H2A	0.9700	C25—C26	1.510 (5)
C2—H2B	0.9700	C25—H25A	0.9700
C3—C4	1.520 (6)	C25—H25B	0.9700
C3—H3A	0.9700	C26—C27	1.524 (5)
C3—H3B	0.9700	C26—H26A	0.9700
C4—C5	1.507 (5)	C26—H26B	0.9700
C4—H4A	0.9700	C27—C28	1.528 (4)
C4—H4B	0.9700	C27—H27A	0.9700
C5—C6	1.516 (4)	C27—H27B	0.9700
C5—H5A	0.9700	C28—N3	1.460 (4)
C5—H5B	0.9700	C28—H28A	0.9800
C6—N2	1.460 (4)	C29—C30	1.501 (5)
C6—H6A	0.9800	C29—H29A	0.9600
C7—C8	1.489 (5)	C29—H29B	0.9600
C7—H7A	0.9600	C29—H29C	0.9600
C7—H7B	0.9600	C30—N3	1.283 (4)
C7—H7C	0.9600	C30—C31	1.474 (5)
C8—N2	1.281 (4)	C31—C36	1.401 (6)
C8—C9	1.488 (5)	C31—C32	1.402 (5)
C9—C10	1.391 (5)	C32—C33	1.370 (6)
C9—C14	1.404 (5)	C32—H32A	0.9300
C10—C11	1.378 (5)	C33—C34	1.372 (8)
C10—H10A	0.9300	C33—H33A	0.9300
C11—C12	1.389 (6)	C34—C35	1.358 (7)
C11—H11A	0.9300	C34—H34A	0.9300
C12—C13	1.366 (6)	C35—C36	1.400 (5)
C12—H12A	0.9300	C35—H35A	0.9300
C13—C14	1.382 (5)	C36—O3	1.336 (5)
C13—H13A	0.9300	C37—C38	1.498 (5)
C14—O2	1.339 (4)	C37—H37A	0.9600
C15—C16	1.498 (5)	C37—H37B	0.9600
C15—H15A	0.9600	C37—H37C	0.9600
C15—H15B	0.9600	C38—N4	1.284 (4)
C15—H15C	0.9600	C38—C39	1.473 (5)
C16—N1	1.287 (4)	C39—C40	1.408 (5)
C16—C17	1.474 (5)	C39—C44	1.411 (5)
C17—C18	1.396 (5)	C40—C41	1.364 (6)
C17—C22	1.415 (6)	C40—H40A	0.9300
C18—C19	1.377 (6)	C41—C42	1.375 (6)
C18—H18A	0.9300	C41—H41A	0.9300
C19—C20	1.403 (8)	C42—C43	1.370 (6)
C19—H19A	0.9300	C42—H42A	0.9300
C20—C21	1.356 (7)	C43—C44	1.387 (5)
C20—H20A	0.9300	C43—H43A	0.9300
C21—C22	1.377 (6)	C44—O4	1.337 (4)
C21—H21A	0.9300	O1—H1	0.8200
C22—O1	1.333 (5)	O2—H2	0.8200
C23—N4	1.462 (4)	O3—H3	0.8200
C23—C24	1.530 (4)	O4—H4	0.8200
			
N1—C1—C2	107.4 (3)	C24—C23—H23A	109.7
N1—C1—C6	110.4 (3)	C28—C23—H23A	109.7
C2—C1—C6	110.7 (3)	C25—C24—C23	111.7 (3)
N1—C1—H1A	109.4	C25—C24—H24A	109.3
C2—C1—H1A	109.4	C23—C24—H24A	109.3
C6—C1—H1A	109.4	C25—C24—H24B	109.3
C3—C2—C1	112.0 (3)	C23—C24—H24B	109.3
C3—C2—H2A	109.2	H24A—C24—H24B	107.9
C1—C2—H2A	109.2	C26—C25—C24	111.7 (3)
C3—C2—H2B	109.2	C26—C25—H25A	109.3
C1—C2—H2B	109.2	C24—C25—H25A	109.3
H2A—C2—H2B	107.9	C26—C25—H25B	109.3
C2—C3—C4	110.7 (4)	C24—C25—H25B	109.3
C2—C3—H3A	109.5	H25A—C25—H25B	108.0
C4—C3—H3A	109.5	C25—C26—C27	111.6 (3)
C2—C3—H3B	109.5	C25—C26—H26A	109.3
C4—C3—H3B	109.5	C27—C26—H26A	109.3
H3A—C3—H3B	108.1	C25—C26—H26B	109.3
C5—C4—C3	110.1 (4)	C27—C26—H26B	109.3
C5—C4—H4A	109.6	H26A—C26—H26B	108.0
C3—C4—H4A	109.6	C26—C27—C28	111.6 (3)
C5—C4—H4B	109.6	C26—C27—H27A	109.3
C3—C4—H4B	109.6	C28—C27—H27A	109.3
H4A—C4—H4B	108.2	C26—C27—H27B	109.3
C4—C5—C6	113.2 (3)	C28—C27—H27B	109.3
C4—C5—H5A	108.9	H27A—C27—H27B	108.0
C6—C5—H5A	108.9	N3—C28—C27	108.5 (3)
C4—C5—H5B	108.9	N3—C28—C23	109.7 (2)
C6—C5—H5B	108.9	C27—C28—C23	110.8 (3)
H5A—C5—H5B	107.7	N3—C28—H28A	109.3
N2—C6—C5	108.2 (3)	C27—C28—H28A	109.3
N2—C6—C1	109.7 (3)	C23—C28—H28A	109.3
C5—C6—C1	110.6 (3)	C30—C29—H29A	109.5
N2—C6—H6A	109.4	C30—C29—H29B	109.5
C5—C6—H6A	109.4	H29A—C29—H29B	109.5
C1—C6—H6A	109.4	C30—C29—H29C	109.5
C8—C7—H7A	109.5	H29A—C29—H29C	109.5
C8—C7—H7B	109.5	H29B—C29—H29C	109.5
H7A—C7—H7B	109.5	N3—C30—C31	116.4 (3)
C8—C7—H7C	109.5	N3—C30—C29	124.6 (3)
H7A—C7—H7C	109.5	C31—C30—C29	119.0 (3)
H7B—C7—H7C	109.5	C36—C31—C32	118.0 (4)
N2—C8—C9	116.7 (3)	C36—C31—C30	120.9 (3)
N2—C8—C7	125.2 (3)	C32—C31—C30	121.1 (4)
C9—C8—C7	118.0 (3)	C33—C32—C31	121.0 (5)
C10—C9—C14	117.5 (3)	C33—C32—H32A	119.5
C10—C9—C8	121.3 (3)	C31—C32—H32A	119.5
C14—C9—C8	121.2 (3)	C32—C33—C34	120.8 (5)
C11—C10—C9	122.7 (4)	C32—C33—H33A	119.6
C11—C10—H10A	118.6	C34—C33—H33A	119.6
C9—C10—H10A	118.6	C35—C34—C33	119.3 (5)
C10—C11—C12	118.5 (4)	C35—C34—H34A	120.3
C10—C11—H11A	120.8	C33—C34—H34A	120.3
C12—C11—H11A	120.8	C34—C35—C36	121.7 (5)
C13—C12—C11	120.1 (4)	C34—C35—H35A	119.1
C13—C12—H12A	119.9	C36—C35—H35A	119.1
C11—C12—H12A	119.9	O3—C36—C35	118.7 (4)
C12—C13—C14	121.5 (4)	O3—C36—C31	122.3 (3)
C12—C13—H13A	119.3	C35—C36—C31	119.0 (4)
C14—C13—H13A	119.3	C38—C37—H37A	109.5
O2—C14—C13	117.9 (3)	C38—C37—H37B	109.5
O2—C14—C9	122.3 (3)	H37A—C37—H37B	109.5
C13—C14—C9	119.7 (4)	C38—C37—H37C	109.5
C16—C15—H15A	109.5	H37A—C37—H37C	109.5
C16—C15—H15B	109.5	H37B—C37—H37C	109.5
H15A—C15—H15B	109.5	N4—C38—C39	116.7 (3)
C16—C15—H15C	109.5	N4—C38—C37	125.0 (3)
H15A—C15—H15C	109.5	C39—C38—C37	118.2 (3)
H15B—C15—H15C	109.5	C40—C39—C44	116.6 (3)
N1—C16—C17	116.1 (3)	C40—C39—C38	122.3 (3)
N1—C16—C15	124.7 (3)	C44—C39—C38	121.1 (3)
C17—C16—C15	119.2 (3)	C41—C40—C39	122.4 (4)
C18—C17—C22	117.4 (4)	C41—C40—H40A	118.8
C18—C17—C16	121.6 (4)	C39—C40—H40A	118.8
C22—C17—C16	120.9 (3)	C40—C41—C42	120.0 (4)
C19—C18—C17	121.9 (5)	C40—C41—H41A	120.0
C19—C18—H18A	119.0	C42—C41—H41A	120.0
C17—C18—H18A	119.0	C43—C42—C41	119.8 (4)
C18—C19—C20	119.4 (5)	C43—C42—H42A	120.1
C18—C19—H19A	120.3	C41—C42—H42A	120.1
C20—C19—H19A	120.3	C42—C43—C44	121.2 (4)
C21—C20—C19	119.1 (5)	C42—C43—H43A	119.4
C21—C20—H20A	120.4	C44—C43—H43A	119.4
C19—C20—H20A	120.4	O4—C44—C43	118.1 (4)
C20—C21—C22	122.4 (5)	O4—C44—C39	121.8 (3)
C20—C21—H21A	118.8	C43—C44—C39	120.1 (3)
C22—C21—H21A	118.8	C16—N1—C1	125.7 (3)
O1—C22—C21	118.2 (4)	C8—N2—C6	125.1 (3)
O1—C22—C17	122.1 (3)	C30—N3—C28	125.3 (3)
C21—C22—C17	119.7 (4)	C38—N4—C23	124.5 (3)
N4—C23—C24	108.3 (2)	C22—O1—H1	109.5
N4—C23—C28	109.1 (3)	C14—O2—H2	109.5
C24—C23—C28	110.3 (3)	C36—O3—H3	109.5
N4—C23—H23A	109.7	C44—O4—H4	109.5
			
C16—N1—C1—C6	102.8 (4)	C38—N4—C23—C28	101.2 (3)
C8—N2—C6—C1	104.3 (4)	N1—C1—C6—N2	−69.1 (3)
C30—N3—C28—C23	100.4 (4)	N3—C28—C23—N4	−65.9 (3)

### Hydrogen-bond geometry (Å, °)

**Table:**

*D*—H···*A*	*D*—H	H···*A*	*D*···*A*	*D*—H···*A*
O1—H1···N1	0.82	1.77	2.496 (4)	147
O2—H2···N2	0.82	1.81	2.531 (4)	147
O3—H3···N3	0.82	1.82	2.507 (4)	140
O4—H4···N4	0.82	1.78	2.507 (4)	147
C26—H26A···Cg3^i^	0.97	2.96	3.790 (5)	144
C29—H29C···Cg3^ii^	0.96	2.96	3.721 (5)	137
C37—H37C···Cg3^iii^	0.96	3.00	3.714 (4)	133

Symmetry codes: (i) *x*, *y*, *z*+1; (ii) −*x*+2, *y*−1/2, −*z*+1; (iii) −*x*+2, *y*+1/2, −*z*+1.
